# Impact of *IL31RA* Genetic Variants and Expression on Metastatic Progression in Oral Cavity Squamous Cell Carcinoma

**DOI:** 10.32604/or.2026.080527

**Published:** 2026-08-13

**Authors:** Hsueh-Ju Lu, Chiao-Wen Lin, Chun-Yi Chuang, Chun-Wen Su, Shun-Fa Yang

**Affiliations:** 1 Division of Hematology and Oncology, Department of Internal Medicine, Chung Shan Medical University Hospital, Taichung, Taiwan; 2 School of Medicine, Chung Shan Medical University, Taichung, Taiwan; 3 Institute of Oral Sciences, Chung Shan Medical University, Taichung, Taiwan; 4 Department of Dentistry, Chung Shan Medical University Hospital, Taichung, Taiwan; 5 Department of Otolaryngology, Chung Shan Medical University Hospital, Taichung, Taiwan; 6 Institute of Medicine, Chung Shan Medical University, Taichung, Taiwan; 7 Department of Medical Research, Chung Shan Medical University Hospital, Taichung, Taiwan

**Keywords:** Interleukin-31 receptor alpha (*IL31RA*), single-nucleotide polymorphism, oral cavity squamous cell carcinoma, migration, lymph node involvement

## Abstract

**Background:** Interleukin-31 receptor alpha (IL31RA) has been implicated in cancer progression and tumor cell migration, but its genetic associations across cancers remain unclear. This study aimed to examine *IL31RA* polymorphisms in relation to lymph node involvement in oral cavity squamous cell carcinoma (OCSCC). **Methods:** In this case-control study, 2845 participants were enrolled, including 1352 patients with OCSCC and 1493 cancer-free controls. Associations between *IL31RA* SNPs and OCSCC susceptibility and clinicopathological characteristics were evaluated. Functional analyses, including cell migration assays, together with bioinformatic database analyses, were performed to investigate the biological significance of IL31RA and genotype–expression correlations. Logistic regression and other appropriate statistical methods were used to assess these associations. **Results:** *IL31RA* polymorphisms did not significantly influence OCSCC development. However, subsite-specific analysis revealed that gingival cancer patients with minor alleles at rs6876491 and rs10055201 had significantly higher lymph node involvement rates than wild-type carriers. *IL31RA* rs6876491 was also an independent predictor of lymph node involvement in gingival cancer after adjusting for clinical characteristics, with adjusted odds ratios of 2.172 (95% Confidence Interval: 1.095–4.310). Bioinformatic databases also showed that minor genotypes correlated with increased IL31RA expression. Patients with higher IL31RA expression tended to have poorer survival in head and neck squamous cell carcinoma (HNSCC) based on TCGA public database analyses (*p* = 0.053). Moreover, functional studies confirmed that IL31RA overexpression enhanced cellular migration (*p* < 0.05), while knockdown suppressed migratory capacity (*p* < 0.05). **Conclusions:** *IL31RA* polymorphisms may be associated with an increased propensity for lymph node involvement, particularly in gingival cancer, suggesting that IL31RA may have potential clinical relevance in OCSCC progression.

## 1 Introduction

Oral cavity squamous cell carcinoma (OCSCC) represents one of the largest subgroups of head and neck squamous cell carcinoma (HNSCC), the eighth most common cancer globally, and the fourth most common cancer among males in Taiwan [1]. Although several novel agents have been reported to improve survival during curative treatment [2], outcomes remain poor once the disease progresses to distant metastasis [3,4]. Despite extensive discussion of pathophysiologic mechanisms in cancer metastasis [5], detailed information regarding OCSCC remains limited. This study aimed to identify possible genetic alterations related to cancer metastasis that may inform future therapeutic strategies.

The metastatic process encompasses dissemination and invasion, intravasation, circulation, extravasation, and colonization [6,7]. During dissemination and invasion, metastatic cancer cells comprise a diverse collection possessing distinct genetic and phenotypic characteristics that differentially drive progression, metastasis, and drug resistance [8]. Several genes have been significantly associated with cancer metastasis, including TP53, CDKN2A, PTEN, PIK3CA, and RB1 [9,10]. However, whether the functions of genetic alterations remain consistent across different cancer types represents an important unresolved question.

Interleukin-31 (IL-31), which binds to a heterodimeric receptor composed of the exclusive interleukin-31 receptor alpha (IL-31RA) chain and the shared oncostatin M receptor (OSMR), is a cytokine associated with skin immunity in previous studies [11]. Several immune cells produce IL-31, and upon binding to its receptor complex—IL31RA/OSMR, IL-31 activates JAK kinases that subsequently phosphorylate and activate STAT1, STAT3, and STAT5. Co-localization with OSMR enables the complex to activate PI3K/AKT and MAPK signaling pathways, including ERK, p38, and JNK [12,13]. IL31RA, the receptor for IL-31 encoded by the IL31RA gene, mediates activation of STAT3 and STAT5, which are significantly related to cancer progression and metastasis [12,13,14]. For instance, IL31RA promotes cancer metastasis by increasing invasion and cancer stem cell-like properties in basal-like breast cancer [14]. Moreover, Aarstad et al. reported that elevated plasma levels of IL-31 predicted overall survival in patients with human papillomavirus (HPV)-negative head and neck squamous cell carcinoma [15]. However, the relationship between the IL-31/IL-31 receptor axis and OCSCC, particularly focusing on IL31RA and cancer metastasis, remains poorly studied.

Single-nucleotide polymorphisms (SNPs) are defined as minor allele frequencies exceeding 1% in at least one population, distinguishing them from rare mutations [16,17]. The human genome contains millions of SNPs distributed across coding and non-coding regions. Although most SNPs are functionally silent, certain variants in genes governing DNA mismatch repair, cell cycle regulation, metabolism, and immunity are associated with cancer susceptibility [18]. Accumulating evidence indicates that SNPs serve as effective biomarkers for predicting cancer progression and metastasis [19]. Notably, IL31RA has been implicated in cancer metastasis across diverse malignancies, rendering it a compelling candidate for investigation in OCSCC pathogenesis [14,20]. Therefore, we focused on IL31RA polymorphisms in OCSCC, particularly regarding cancer metastasis.

We conducted a retrospective case-control study enrolling patients diagnosed with OCSCC and healthy controls. The impact of IL31RA polymorphisms on cancer progression was analyzed based on our cohort. Functional validation assays and published bioinformatic databases were utilized to verify our findings. This study aims to enhance understanding of the roles of IL31RA polymorphisms in cancer progression, particularly in OCSCC.

## 2 Materials and Methods

### 2.1 Study Subjects

This retrospective case-control study enrolled patients diagnosed with oral cavity squamous cell carcinoma (OCSCC) between 2007 and 2022 at Chung Shan Medical University Hospital as the case group. Inclusion criteria for the case group were: (1) histopathologically confirmed OCSCC, (2) age ≥ 20 years, and (3) availability of complete clinical and blood specimen data. Exclusion criteria included: (1) absence of pathological confirmation, (2) history of second primary malignancy. For the case group, basic characteristics and personal habits were recorded, including age, cigarette smoking, alcohol consumption, and betel quid chewing. Pathological factors were also collected as described in our previous studies [21]. The control group comprised healthy participants aged 30–70 years with normal mental capacity and no cancer history, recruited from Taiwan Biobank. Blood specimens from both groups were collected for analysis. The study was approved by the Institutional Review Board of Chung Shan Medical University Hospital (CSMUH No.: CS1-21151). We confirm that the study was conducted in accordance with the Declaration of Helsinki and that written informed consent was obtained from all participants.

### 2.2 DNA Extraction and Genotyping

Whole-blood specimens were collected in sterile tubes containing ethylenediaminetetraacetic acid (EDTA) and immediately centrifuged at 3000 rpm for 5 min using a KUBOTA S300T centrifuge (Kubota Corporation, Tokyo, Japan). The buffy coat (leukocyte fraction) was isolated and stored at −80°C. Genomic DNA was extracted from peripheral blood leukocytes using the QIAamp DNA Blood Mini Kit. (Cat. no. 51104. (Qiagen, Valencia, CA, USA). According to the manufacturer’s protocol. Genomic DNA was dissolved in TE buffer (10 mM Tris-HCl and 1 mM EDTA; pH 7.8) and quantified by measuring optical density at 260 nm using a NanoDrop 2000 spectrophotometer (Thermo Fisher Scientific, Waltham, MA, USA). The final product was stored at −20°C and used as a template for polymerase chain reaction (PCR). The impact of IL31RA polymorphisms rs6876491, rs1195646, and rs10055201 on cancer progression was investigated in our cohort. Genotyping was performed using TaqMan assays, with results analyzed using SDS version 3.0 software (Applied Biosystems, Foster City, CA, USA). Each 10-μL reaction mixture contained 5 μL of Genotyping Master Mix, 0.25 μL of TaqMan probe, 1 μL of genomic DNA template (10 ng/μL; 10 ng total), and 3.75 μL of nuclease-free water. Real-time PCR conditions consisted of an initial denaturation step at 95°C for 10 min, followed by 40 amplification cycles of 95°C for 15 s and 60°C for 1 min. 

### 2.3 Cells and Cell Culture

The oral cancer cell lines Ca9-22 (cat. no. RCB1976; RIKEN BioResource Research Center, Tsukuba, Ibaraki, Japan), HSC-3 (cat. no. JCRB0623; Japanese Collection of Research Bioresources Cell Bank, Osaka, Japan), SAS (JCRB cell line; Japanese Collection of Research Bioresources Cell Bank, Osaka, Japan), SCC-9 (cat. no. CRL-1629; American Type Culture Collection [ATCC], Manassas, VA, USA), and TW2.6 (human oral squamous cell carcinoma cell line, established at National Taiwan University Hospital, Taipei, Taiwan) were used in this study. Ca9-22 cells were maintained in Minimum Essential Medium (MEM; cat. no. 11095-080, Gibco, Thermo Fisher Scientific Inc., Waltham, MA, USA) supplemented with 10% fetal bovine serum (FBS). SCC-9 cells were cultured in DMEM/F-12 medium (1:1; cat. no. 11320-033, Gibco, Thermo Fisher Scientific Inc., Waltham, MA, USA) supplemented with 10% FBS and 400 ng/mL hydrocortisone (cat. no. H0888, Sigma-Aldrich, St. Louis, MO, USA). HSC-3 cells were maintained in Dulbecco’s Modified Eagle Medium (DMEM; cat. no. 11965-092, Gibco, Thermo Fisher Scientific Inc., Waltham, MA, USA) supplemented with 10% FBS. SAS cells were cultured in DMEM supplemented with 10% FBS, and TW2.6 cells were maintained in RPMI-1640 medium (cat. no. 11875-093, Gibco, Thermo Fisher Scientific Inc., Waltham, MA, USA), each under standard cell culture conditions at 37°C in a humidified atmosphere containing 5% CO_2_. Cells were kept at 37°C in a humidified incubator with 5% CO_2_, following standard culture conditions. All cell lines are short tandem repeat (STR) authenticated. Mycoplasma contamination was routinely tested by PCR analysis.

### 2.4 IL31RA Overexpression Vector and siRNA Transfection

Ca9-22 and SCC-9 cells were seeded in 6-cm dishes at a density of 4 × 10^5^ cells per dish and cultured at 37°C in a humidified atmosphere containing 5% CO_2_. Cells reached approximately 70–80% confluence after 16 h and were then transfected with the indicated siRNAs. After 42–48 h of incubation, the transfected cells were harvested, counted, and re-seeded at the appropriate density for subsequent migration assays. Cell viability was confirmed by trypan blue exclusion before each assay. For IL31RA overexpression, 2.5 μg of either the pcDNA3.1(+) control vector and pcDNA3.1(+)-IL31RA expression vector (Thermo Fisher Scientific Inc., Waltham, Massachusetts, USA) construct was mixed with 2.5 μL of Lipofectamine 2000 Transfection Reagent (cat. no. 11668019, Thermo Fisher Scientific Inc., Waltham, MA, USA) for 20 min and added to cells in Opti-MEM. After 6 h, the medium was replaced with standard growth medium, and cells were harvested 42 h later for mRNA and migration analyses. For IL31RA knockdown, cells were transfected with 10 nM negative-control or two different IL31RA-specific siRNAs (GenePharma, Shanghai, China) using Lipofectamine RNAiMAX (cat. no. 13778150, Thermo Fisher Scientific Inc., Waltham, MA, USA). The siRNA sequences were as follows: negative-control siRNA sense, 5′-UUCUCCGAACGUGUCACGUTT-3′, and antisense, 5′-ACGUGACACGUUCGGAGAATT-3′; IL31RA siRNA-1 sense, 5′-CCCUCACUCUGCAAAUUCATT-3′, and antisense, 5′-UGAAUUUGCAGAGUGAGGGTT-3′; IL31RA siRNA-2 sense, 5′-GCCUGUUUCAUCUGAUUUATT-3′, and antisense, 5′-UAAAUCAGAUGAAACAGGCTT-3′. The siRNA and Lipofectamine RNAiMAX were mixed at a 1:1 (v/v; μL:μL) ratio and incubated for 20 min at room temperature to allow complex formation prior to transfection. Cells were collected 48 h after transfection for expression profiling and phenotypic assays.

### 2.5 Transwell Migration Assay

Cell migratory capacity was evaluated using a Transwell migration system (Millicell™ Cell Culture Inserts, 8 μm pore size, MilliporeSigma, Burlington, MA, USA). A total of 3 × 10^4^ cells in serum-free medium were seeded into the upper chamber, while medium containing 10% fetal bovine serum was added to the lower chamber as a chemoattractant [5]. For the assay, the lower chamber was filled with complete medium containing 10% FBS as a chemoattractant. Ca9-22 and SCC-9 cells suspended in serum-free medium were seeded into the upper chamber. After a 48-h incubation at 37°C, the membranes were then fixed in methanol for 10 min and stained with 10% Giemsa solution (cat. no. GS500, Sigma-Aldrich, St. Louis, MO, USA) for 3 h at room temperature. After washing with distilled water and air-drying, the migrated cells on the lower surface of the membrane were observed and quantified under a microscope (IX73, Olympus Corporation, Tokyo, Japan).

### 2.6 Published Databases for Validation

Multiple published databases were utilized to validate our findings. The Genotype-Tissue Expression (GTEx) portal provides comprehensive data on human gene expression, regulation, and genetic variation across diverse tissues, incorporating whole-genome sequencing, whole-exome sequencing, and RNA-seq analyses (https://gtexportal.org/home/) [22]. The UALCAN database offers an interactive platform for analyzing cancer OMICS data from TCGA, MET500, CPTAC, and CBTTC (https://ualcan.path.uab.edu/) [23]. The Human Protein Atlas contains genome-wide RNA expression data correlated with survival outcomes from 8384 cancer patients across 21 cancer types, plus immunohistochemistry (IHC)-based protein expression patterns from 216 tumors representing the 20 most common cancers (https://www.proteinatlas.org/) [24]. Finally, cBioPortal provides open-access exploration of multidimensional cancer clinical information and genomics datasets, including TCGA (https://www.cbioportal.org/) [25]. For the purposes of this study, the data extracted and analyzed from the UALCAN database, the Human Protein Atlas, and cBioPortal were derived from TCGA cohort datasets available through these platforms.

### 2.7 Statistical Analysis

Clinicopathological parameters were compared using χ^2^ and Fisher’s exact tests. Univariate and multivariate logistic regression analyses were performed to identify independent risk factors for cancer development and nodal involvement, respectively, with results expressed as odds ratios (ORs) with 95% confidence intervals (CIs). Adjusted odds ratios (AORs) indicate that the effect of the IL31RA allele was adjusted for age, sex, cigarette smoking, alcohol consumption, and betel quid chewing in the cancer development model. In the nodal involvement model, the effect was further adjusted for other clinicopathological parameters. Survival outcomes were assessed using Kaplan-Meier plots and log-rank tests. Statistical significance was set at *p* < 0.05 (two-sided). All analyses were conducted using SPSS version 21.0 (SPSS Inc., Chicago, IL, USA).

## 3 Results

### 3.1 Baseline Characteristics

A total of 2845 participants were enrolled, comprising 1352 cases (47.5%) and 1493 controls. OCSCC patients differed significantly from healthy controls in age, sex, and personal habits (all *p* < 0.05). Among cases, 54.4% (735/1352) presented with advanced stage disease (stage III/IV), 32.0% (432/1352) exhibited regional nodal metastasis, and 84.2% (1138/1352) demonstrated moderate to poor histologic differentiation (Table 1).

**Table 1:** Basic characteristics of the patients with oral cancer and healthy controls.

Variable	Controls (N = 1493)	Patients (N = 1352)	*p* Value
Age (years) ≥55 <55	787 (52.7%) 706 (47.3%)	792 (58.6%) 560 (41.4%)	0.001
Sex Male Female	1194 (80.0%) 299 (20.0%)	1289 (95.3%) 63 (4.7%)	<0.001
Cigarette smoking Yes No	645 (43.2%) 848 (56.8%)	1037 (76.7%) 315 (23.3%)	<0.001
Alcohol drinking Yes No	242 (16.2%) 1251 (83.8%)	516 (38.2%) 836 (61.8%)	<0.001
Betel quid chewing Yes No	198 (13.3%) 1295 (86.7%)	902 (66.7%) 450 (33.3%)	<0.001
Pathologic staging I + II III + IV	NA	617 (45.6%) 735 (54.4%)	NA
Pathologic T staging T1 + T2 T3 + T4	NA	650 (48.1%) 702 (51.9%)	NA
Pathologic N staging N0 N+	NA	920 (68.0%) 432 (32.0%)	NA
Pathologic M staging M0 M1	NA	1345 (99.5%) 7 (0.5%)	NA
Histological differentiation Well Moderate to poor	NA	214 (15.8%) 1138 (84.2%)	NA

Note: *p*-values were calculated using the Chi-squared test or Fisher’s exact test, as appropriate for the categorical variable. NA, not applicable.

### 3.2 Relationship between IL31RA Polymorphisms and OCSCC Development

The frequencies of individuals carrying at least one variant allele (CT + TT for rs6876491, TC + CC for rs1195646, and AG + GG for rs10055201) were 68.5% (1949/2845), 75.6% (2152/2845), and 72.0% (2047/2845), respectively, in the total cohort. In the case group, the corresponding frequencies were 67.9% (918/1352), 76.0% (1028/1352), and 71.7% (970/1352). No significant differences in variant rates were observed between cases and controls. After adjusting for age, sex, cigarette smoking, alcohol consumption, and betel quid chewing, the minor alleles did not significantly impact OCSCC development in logistic regression, indicating these polymorphisms do not influence OCSCC susceptibility (Table 2).

**Table 2:** Odds ratios (OR) and 95% confidence interval (CI) of oral cancer associated with IL31RA genotypic frequencies.

Variable	Patients (N, %)	Controls (N, %)	*p* Value	OR (95% CI)	AOR (95% CI)^a^
	N = 1352	N = 1493			
rs6876491 CC CT TT CT + TT	434 (32.1%) 688 (50.9%) 230 (17.0%) 918 (67.9%)	462 (30.9%) 743 (49.8%) 288 (19.3%) 1031 (69.1%)	0.286	1 0.986 (0.834–1.165) 0.850 (0.684–1.056) 0.948 (0.809–1.110)	1 0.997 (0.814–1.220) 0.824 (0.630–1.076) 0.949 (0.784–1.149)
rs1195646 TT TC CC TC + CC	324 (24.0%) 715 (52.9%) 313 (23.1%) 1028 (76.0%)	369 (24.7%) 754 (50.5%) 370 (24.8%) 1124 (75.3%)	0.421	1 1.080 (0.901–1.294) 0.963 (0.779–1.191) 1.042 (0.877–1.237)	1 1.136 (0.911–1.416) 1.015 (0.785–1.313) 1.096 (0.890–1.349)
rs10055201 AA AG GG AG + GG	382 (28.3%) 702 (51.9%) 268 (19.8%) 970 (71.7%)	416 (27.9%) 756 (50.6%) 321 (21.5%) 1077 (72.1%)	0.540	1 1.011 (0.851–1.202) 0.909 (0.734–1.126) 0.981 (0.833–1.155)	1 0.973 (0.789–1.200) 0.863 (0.664–1.122) 0.943 (0.774–1.149)

Note: OR, odds ratio; CI, confidence interval; AOR, adjusted odds ratio. ^a^Adjusted for the effects of age, sex, cigarette smoking, alcohol consumption, and betel quid chewing.

### 3.3 Clinicopathological Impact of IL31RA Polymorphisms in OCSCC

The distribution of baseline characteristics was comparable between OCSCC patients with and without variant genotypes in the entire cohort (N = 1352), and the results showed that patients with or without allele mutations were not significantly different (Table 3). However, subgroup analysis of the gingival (N = 201) revealed significant associations. Patients harboring minor alleles rs6876491 demonstrated more advanced staging compared to wild-type carriers: pathologic stage III/IV (75.2% vs. 58.3%, *p* = 0.011), pT3/4 (75.2% vs. 58.3%, *p* = 0.011), and pN+ (36.4% vs. 22.2%, *p* = 0.026). Similarly, rs10055201 minor allele carriers also exhibited higher rates of advanced disease: pathologic stage III/IV (73.3% vs. 60.6%, *p* = 0.048), pT3/4 (73.3% vs. 60.6%, *p* = 0.048) and pN+ (35.6% vs. 22.7%, *p* = 0.045) (Table 4). In contrast, no significant associations were observed between the rs1195646 polymorphism and any clinicopathological characteristics, including pathological stage, tumor size, lymph node status, or cell differentiation (Table 4).

**Table 3:** The distributions of demographical characteristics of IL31RA allele mutation in all OCSCC patients (N = 1352).

Variable	rs6876491			rs1195646			rs10055201		
	CC N = 434 (%)	CT + TT N = 918 (%)	*p* Value	TT N = 324 (%)	TC + CC N = 1028 (%)	*p* Value	AA N = 382 (%)	AG + GG N = 970 (%)	*p* Value
Age >= 55	243 (56.0%)	549 (59.8%)	0.102	191 (59%)	601 (58.5%)	0.465	211 (55.2%)	581 (59.9%)	0.066
Sex Male Female	411 (94.7%) 23 (5.3%)	878 (95.6%) 40 (4.4%)	0.262	310 (95.7%) 14 (4.3%)	979 (95.2%) 49 (4.8%)	0.438	361 (94.5%) 21 (5.5%)	928 (95.7%) 42 (4.3%)	0.217
Personal history cigarette smoking alcohol drinking betel quid chewing	334 (77.0%) 171 (39.4%) 300 (69.1%)	703 (76.6%) 345 (37.6%) 602 (65.6%)	0.468 0.280 0.109	258 (79.6%) 125 (38.6%) 224 (69.1%)	779 (95.8%) 391 (38.0%) 678 (66.0%)	0.087 0.455 0.161	292 (76.4%) 147 (38.5%) 261 (68.3%)	745 (76.8%) 369 (38.0%) 641 (66.1%)	0.469 0.464 0.235
Primary site location cheek mucosa oral tongue gingival lip hard palate mouth floor retromolar trigone others	133 (30.6%) 141 (32.5%) 72 (16.6%) 23 (5.3%) 14 (3.2%) 15 (3.5%) 16 (3.7%) 15 (3.5%)	297 (32.4%) 296 (32.2%) 129 (14.1%) 53 (5.8%) 32 (3.5%) 30 (3.3%) 34 (3.7%) 29 (3.2%)	0.936	95 (29.3%) 107 (33.0%) 46 (14.2%) 21 (6.5%) 12 (3.7%) 12 (3.7%) 14 (4.3%) 12 (3.7%)	335 (32.6%) 330 (32.1%) 155 (15.1%) 55 (5.4%) 34 (3.3%) 33 (3.2%) 36 (3.5%) 32 (3.1%)	0.954	155 (30.1%) 126 (33.0%) 66 (17.3%) 22 (5.8%) 12 (3.1%) 10 (2.6%) 12 (3.1%) 14 (3.7%)	315 (32.5%) 311 (32.1%) 135 (13.9%) 54 (5.6%) 34 (3.5%) 35 (3.6%) 38 (3.9%) 30 (3.1%)	0.782
Pathologic staging Stage I + II Stage III + IV	195 (44.9%) 239 (55.1%)	422 (46.0%) 496 (54.0%)	0.383	154 (47.5%) 170 (52.5%)	463 (45.0%) 565 (55.0%)	0.235	173 (45.3%) 209 (54.7%)	444 (45.8%) 526 (54.2%)	0.460
Pathologic T staging T1/2 T3/4	211 (48.6%) 223 (51.4%)	439 (47.8%) 479 (52.2%)	0.415	164 (50.6%) 160 (49.4%)	486 (47.3%) 542 (52.7%)	0.162	184 (48.2%) 198 (51.8%)	466 (48.0%) 504 (52.0%)	0.507
Pathologic N staging N0 N+	286 (65.9%) 148 (34.1%)	634 (69.1%) 284 (30.9%)	0.135	221 (68.2%) 103 (31.8%)	669 (68.0%) 329 (32.0%)	0.500	257 (67.3%) 125 (32.7%)	663 (68.4%) 307 (31.6%)	0.375
Pathologic M staging M0 M1	432 (99.5%) 2 (0.5%)	913 (99.5%) 5 (0.5%)	0.599	323 (99.7%) 1 (0.3%)	1022 (99.4%) 6 (0.6%)	0.471	381 (99.7%) 1 (0.3%)	964 (99.4%) 6 (0.6%)	0.367
Histological differentiation Well Moderate or poor	70 (16.1%) 364 (83.9%)	144 (15.7%) 774 (84.3%)	0.446	46 (14.2%) 278 (85.8%)	168 (16.3%) 860 (83.7%)	0.203	66 (17.3%) 316 (82.7%)	148 (15.3%) 822 (84.7%)	0.202

**Table 4:** The distributions of demographical characteristics of IL31RA allele mutation in patients with primary site of gingival (N = 201).

Variable	rs6876491			rs1195646			rs10055201		
	CC N = 72 (%)	CT + TT N = 129 (%)	*p* Value	TT N = 46 (%)	TC + CC N = 155 (%)	*p* Value	AA N = 66 (%)	AG + GG N = 135 (%)	*p* Value
Age >= 55	47 (65.3%)	91 (70.5%)	0.269	33 (71.7%)	105 (67.7%)	0.374	43 (65.2%)	95 (70.4%)	0.277
Sex Male Female	66 (91.7%) 6 (8.3%)	119 (92.2%) 10 (7.8%)	0.541	42 (91.3%) 4 (8.7%)	143 (92.3%) 12 (7.7%)	0.520	60 (90.9%) 6 (9.1%)	125 (92.6%) 10 (7.4%)	0.435
Personal history cigarette smoking alcohol drinking betel quid chewing	53 (73.6%) 23 (31.9%) 48 (66.7%)	95 (73.6%) 50 (38.8%) 83 (64.3%)	0.561 0.209 0.431	39 (84.8%) 18 (39.1%) 31 (67.4%)	109 (70.3%) 55 (35.5%) 100 (64.5%)	0.035 0.388 0.431	48 (72.7%) 20 (30.3%) 44 (66.7%)	100 (74.1%) 53 (39.3%) 87 (64.4%)	0.483 0.139 0.441
Pathologic staging Stage I + II Stage III + IV	30 (41.7%) 42 (58.3%)	32 (24.8%) 97 (75.2%)	0.011	12 (26.1%) 34 (73.9%)	50 (32.3%) 105 (67.7%)	0.272	26 (39.4%) 40 (60.6%)	36 (26.7%) 99 (73.3%)	0.048
Pathologic T staging T1/2 T3/4	30 (41.7%) 42 (58.3%)	32 (24.8%) 97 (75.2%)	0.011	11 (23.9%) 35 (76.1%)	51 (32.9%) 104 (67.1%)	0.164	26 (39.4%) 40 (60.6%)	36 (26.7%) 99 (73.3%)	0.048
Pathologic N staging N0 N+	56 (77.8%) 16 (22.2%)	82 (63.6%) 47 (36.4%)	0.026	30 (65.2%) 16 (34.8%)	108 (69.7%) 47 (30.3%)	0.344	51 (77.3%) 15 (22.7%)	87 (64.4%) 48 (35.6%)	0.045
Pathologic M staging M0 M1	71 (98.6%) 1 (1.4%)	128 (99.2%) 1 (0.8%)	0.589	46 (100.0%) 0 (0.0%)	153 (98.7%) 2 (1.3%)	0.594	66 (100.0%) 0 (0.0%)	133 (98.5%) 2 (1.5%)	0.450
Histological differentiation Well Moderate or poor	15 (20.8%) 57 (79.2%)	22 (17.1%) 107 (82.9%)	0.315	10 (21.7%) 36 (78.3%)	27 (17.4%) 128 (82.6%)	0.320	14 (21.2%) 52 (78.8%)	23 (17.0%) 112 (83.0%)	0.297

Note: The meaning of bolded *p*-values has been clarified as indicating statistical significance (*p* < 0.05).

### 3.4 Association of IL31RA Polymorphisms with Lymph Node Involvement

**Figure 1:**
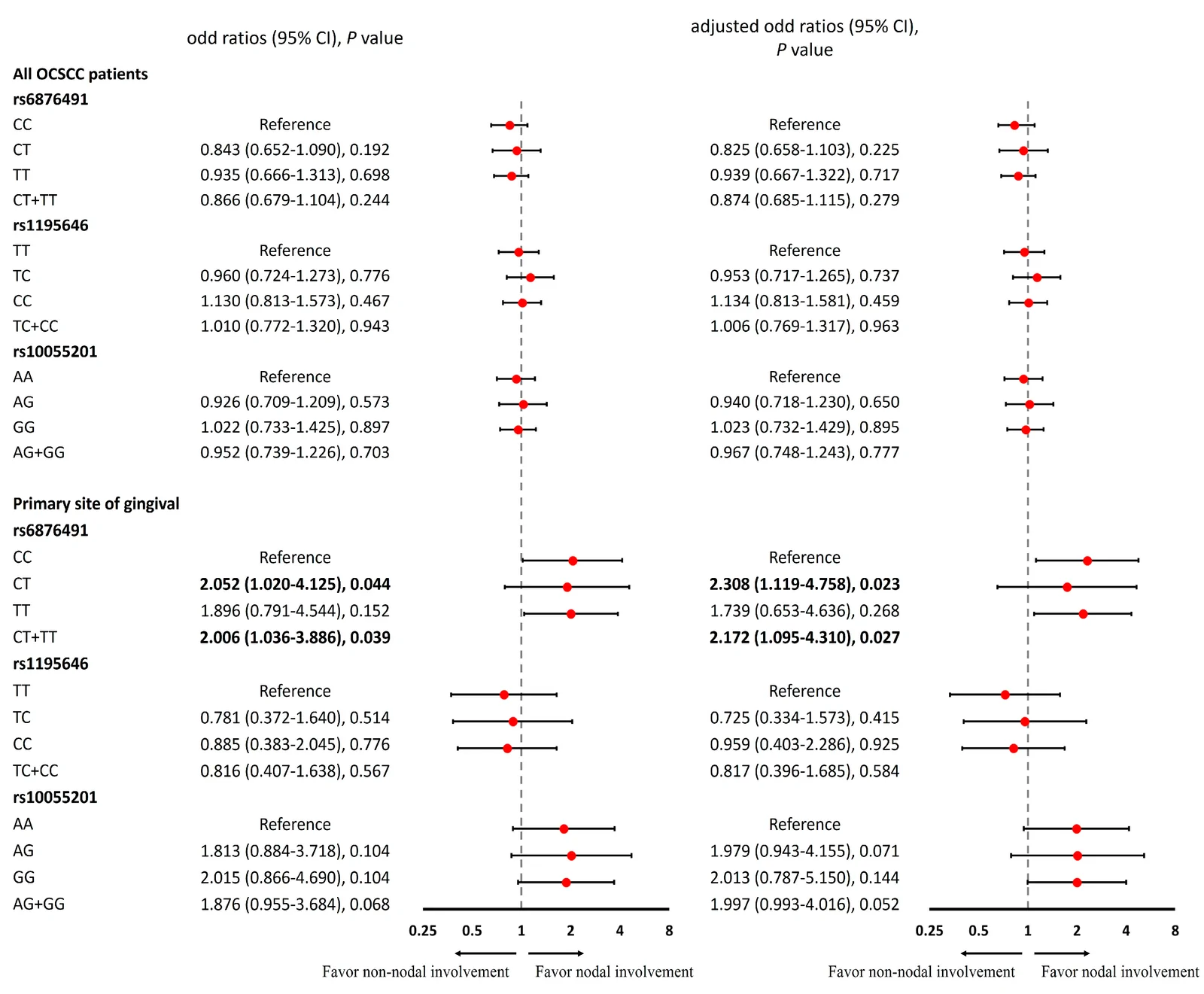
Association of IL31RA polymorphisms with lymph node involvement. Univariate and multivariate logistic regression analyses of IL31RA rs6876491 in all OCSCC patients. Subgroup analysis in gingival cancer patients showed significant associations between minor genotypes and increased lymph node involvement risk.

Univariate and multivariate logistic regression analyses were performed to evaluate the relationships between *IL31RA* polymorphisms and cancer metastasis, such as nodal involvement. Among all OCSCC patients, *IL31RA* polymorphisms showed no significant association with lymph node involvement. However, subgroup analysis of patients with primary gingival tumors revealed significant associations. The *IL31RA* rs6876491 polymorphism (genotype CT + TT versus CC) demonstrated an OR (95% CI) of 2.006 (1.036–3.886). After adjusting for age, sex, cigarette smoking, alcohol drinking, and betel quid chewing, these associations remained significant, with an AOR of 2.172 (1.095–4.310) (Fig. 1). These findings suggest that *IL31RA* rs6876491 genotypes may contribute to lymph node involvement, particularly in gingival cancer patients.

### 3.5 Validation Using Bioinformatic Databases

**Figure 2:**
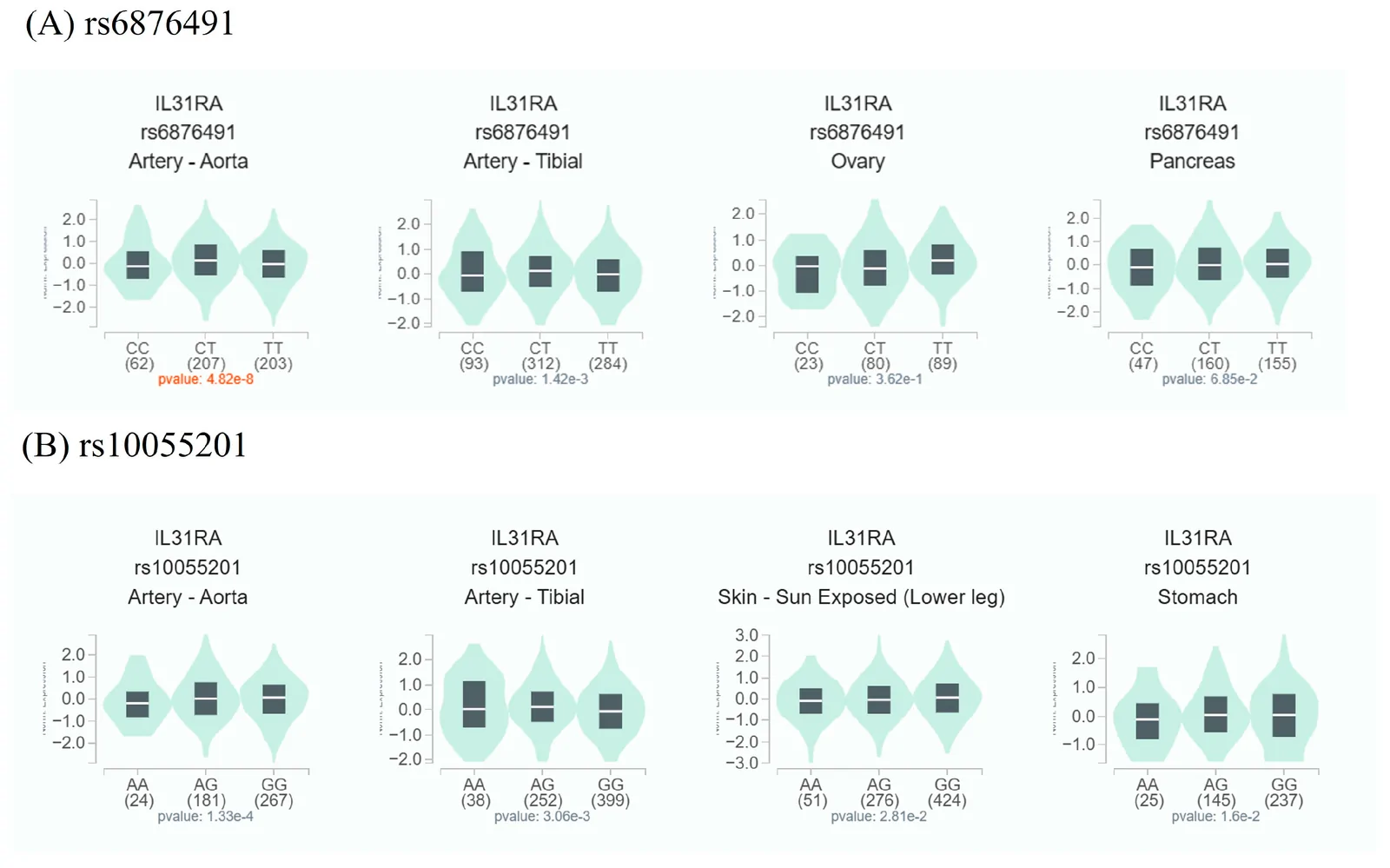
IL31RA expression associated with IL31RA polymorphisms in GTEx. (**A**) IL31RA mRNA expression levels for rs6876491 genotypes across tissues. (**B**) IL31RA mRNA expression levels for rs10055201 genotypes, with minor alleles showing higher expression.

To validate the functional relevance of IL31RA variants, we examined publicly available bioinformatic databases. GTEx database analysis revealed that carriers of the minor/variant alleles rs6876491 and rs10055201 exhibited significantly higher IL31RA expression levels compared with wild-type carriers in specific normal tissues, including arteries, skin, and stomach (Fig. 2). However, no statistically significant differences were observed in other tissues, such as the ovary (*p* = 0.362) and pancreas (*p* = 0.0685).

**Figure 3:**
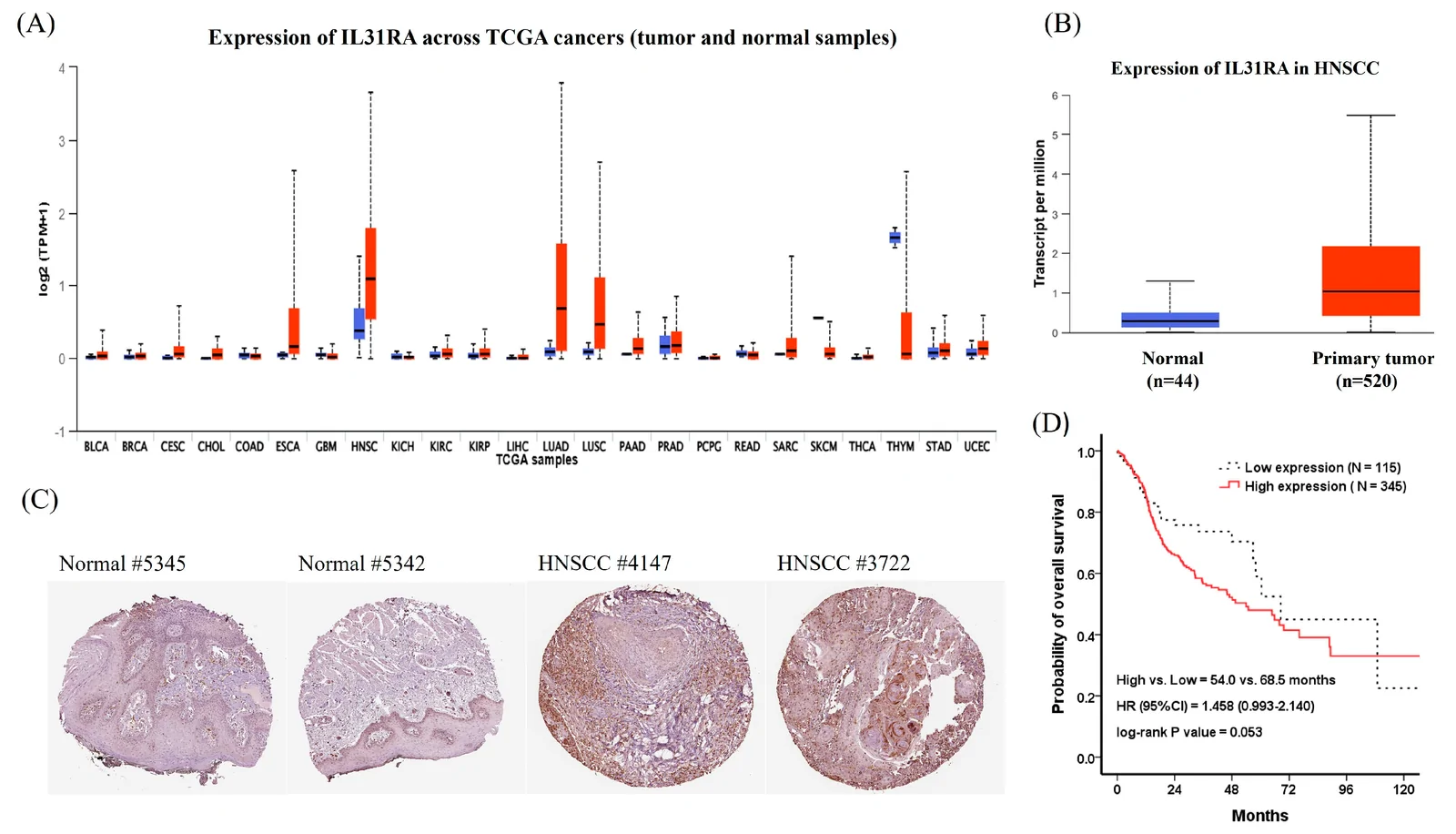
IL31RA expression profiles and prognostic relevance. (**A**,**B**) UALCAN analysis showing elevated IL31RA expression in multiple cancer types compared with normal tissues (*p* = 1.62 × 10^−^^12^). (**C**) Human Protein Atlas IHC confirming higher IL31RA protein levels in tumors. The IHC images were obtained from the HPA database. As no scale bar information is available in the original HPA images, the approximate diameter of the tissue microarray cores (approximately 1 mm) is provided as a spatial reference. (**D**) Kaplan–Meier survival curve based on TCGA head and neck squamous cell carcinoma (HNSCC) dataset, indicating a trend toward poorer overall survival in patients with high IL31RA expression.

According to the UALCAN database, IL31RA expression was elevated in tumor tissues compared with normal tissues across multiple cancer types, including esophageal squamous cell carcinoma, head and neck squamous cell carcinoma (HNSCC), lung adenocarcinoma, and lung squamous cell carcinoma (Fig. 3A,B). In the Human Protein Atlas, IHC staining using antibody HPA068114 also confirmed that IL31RA expression in tumor tissues was relatively enhanced compared to normal tissues in protein level (Fig. 3C). For HNSCC patients, high IL31RA mRNA expression showed a trend toward reduced survival but did not reach statistical significance. Median overall survival was 54.0 months in the high-expression group versus 68.5 months in the low-expression group, with a hazard ratio of 1.458 (95% CI: 0.993–2.140) (Fig. 3D).

### 3.6 Functional Validation Studies

**Figure 4:**
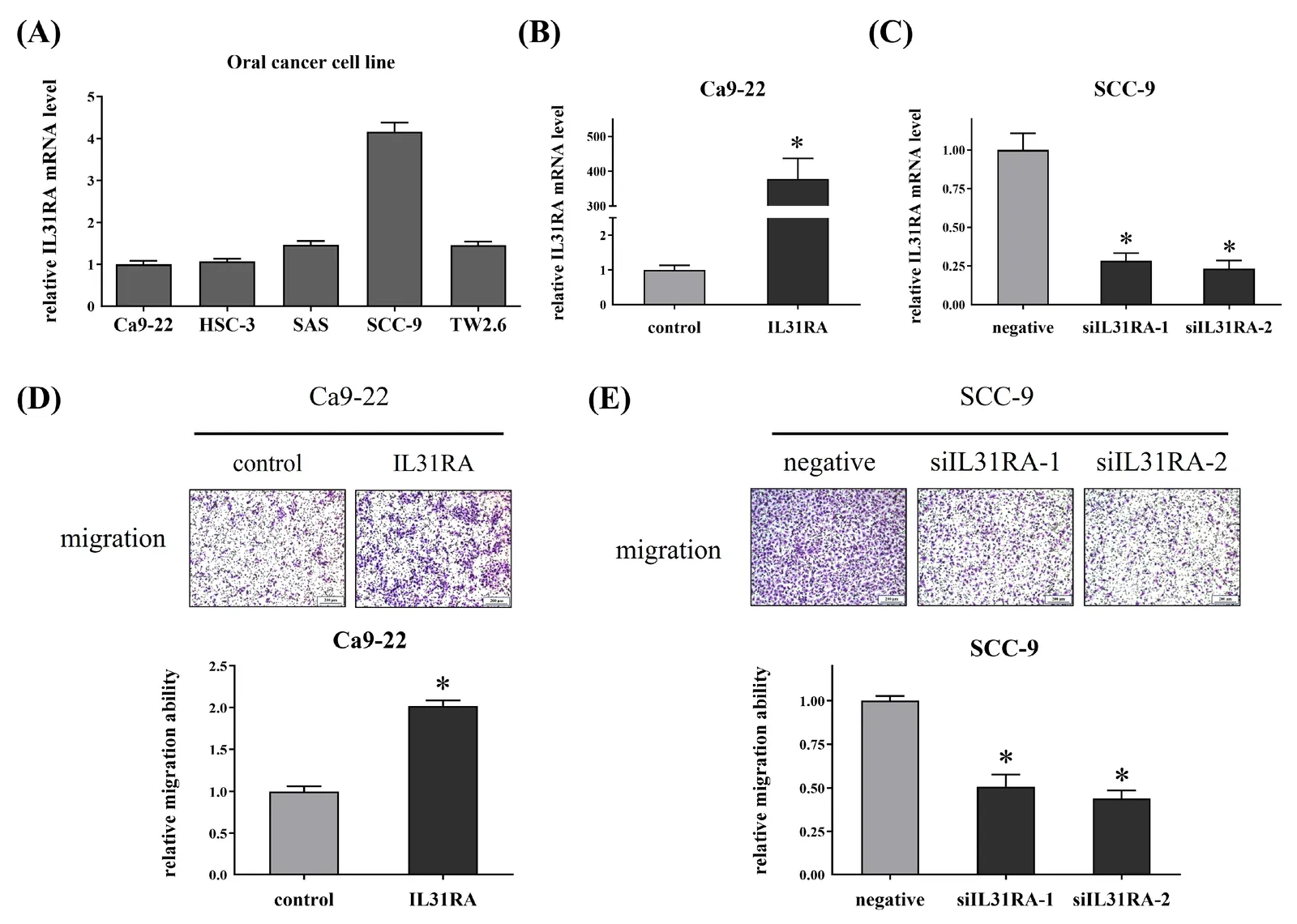
Functional validation of IL31RA in oral cancer cells. (**A**) Endogenous IL31RA expression in oral cancer cell lines. IL31RA mRNA expression levels in Ca9-22, HSC-3, SAS, SCC-9, and TW2.6 cells were analyzed by real-time PCR. (**B**) The IL31RA mRNA level after transfection of the IL31RA overexpression vector in Ca9-22 cell lines. *p < 0.05 compared with control vector. (**C**) The IL31RA mRNA level after transfection of siIL31RA-1 and siIL31RA-2 in SCC-9 cell lines. *p < 0.05 compared with negative siRNA. (**D**) The migration ability after transfection of the IL31RA overexpression vector was analyzed by the transwell assay. *p < 0.05 compared with control vector. (**E**) The migration ability after transfection of IL31RA siRNA was analyzed by the transwell assay. *p < 0.05 compared with negative siRNA.

*In vitro* functional validation was conducted using oral cancer cell lines. We first analyzed IL31RA mRNA expression in five oral cancer cell lines (Ca9-22, HSC-3, SAS, SCC-9, and TW2.6) using real-time PCR assay. Among these cell lines, SCC-9 exhibited the highest IL31RA expression, whereas Ca9-22 showed relatively low expression levels (Fig. 4A). Based on these findings, SCC-9 cells were selected for IL31RA knockdown experiments, while Ca9-22 cells were used for IL31RA overexpression assays. As shown in Fig. 4B,C, IL31RA overexpression in Ca9-22 cells resulted in elevated mRNA expression levels, while siRNA-mediated IL31RA knockdown in SCC-9 cells reduced expression. Migration assays demonstrated that IL31RA overexpression enhanced Ca9-22 cell migration (Fig. 4D), whereas IL31RA knockdown suppressed SCC-9 cell migration (Fig. 4E). These results indicate that oral cancer cell migratory capacity is positively associated with IL31RA expression levels.

## 4 Discussion

This retrospective case-control study included 2845 participants, including 1352 OCSCC patients. IL31RA polymorphisms were not associated with OCSCC risk; however, rs6876491 was significantly associated with lymph node involvement after adjustment for clinical factors. Functional assays and bioinformatic analyses supported a role of IL31RA in tumor progression. IL31RA expression was consistently upregulated in HNSCC, and higher expression was associated with poorer survival. *In vitro* results further showed that IL31RA promoted cancer cell migration, whereas knockdown suppressed this effect. Collectively, IL31RA rs6876491 may contribute to OCSCC progression by enhancing tumor cell migration and lymph node involvement.

Our study has several strengths. First, the large cohort provided robust statistical power to evaluate *IL31RA* polymorphisms in cancer development. Second, convergent evidence from functional assays and bioinformatic analyses supports our genetic findings, suggesting that IL31RA variant alleles may be associated with clinicopathological outcomes specifically in the gingival cancer subpopulation. Third, previous studies have demonstrated that IL31RA expression is involved in cancer progression across various malignancies [14,26], supporting the biological plausibility of our findings. Although the role of *IL31RA* in OCSCC remains unclear, our findings align with its phenotypic effects in other cancers.

Metastasis remains the leading cause of cancer-related mortality. Over the past decade, high-throughput technologies have enabled genome-wide characterization of transcriptomic alterations associated with cancer metastasis [27]. To facilitate a comprehensive analysis of metastatic transcriptome data, integrated databases specifically for metastasis research have also been established [27]. Furthermore, single-cell sequencing studies have elucidated potential pathophysiological mechanisms underlying metastasis in HNSCC [28]. Multiple genetic hypotheses have been proposed to explain the molecular basis of metastatic dissemination, establishing genetic analysis as an indispensable approach in metastasis research [29]. Accumulating evidence demonstrates that SNPs serve as effective biomarkers for predicting cancer progression and metastatic potential [21]. Consequently, these results extend previous evidence on SNPs as potential biomarkers of cancer metastasis by identifying rs6876491 as being associated with lymph node involvement in gingival cancer.

IL31RA, also known as gp130-like monocyte receptor (GLM-R), is encoded by the *IL31RA* gene located on human chromosome 5 [26]. Upon IL31 cytokine binding to IL31RA, the receptor activates JAK1/JAK2, which phosphorylates downstream tyrosine residues on IL31RA. This phosphorylation stimulates STAT family members, mediating proliferation in various cell types, including lung epithelial cells, intestinal epithelial cells, and skin cells [26,30]. Previous studies have reported higher IL31RA expression in malignant specimens compared to healthy controls [12,20]. Supporting a pro-tumorigenic role, He et al. demonstrated that IL31RA overexpression in luminal breast cancer cells enhances cancer stem cell-like properties and cell motility. Conversely, *IL31RA* silencing suppresses cancer stem cell characteristics, migration, and invasion *in vitro*, as well as tumor growth and metastasis *in vivo* [14]. Additionally, Ferretti et al. showed that IL31 enhances cancer proliferation in follicular lymphoma through IL31RA-driven STAT, ERK1/2, and AKT phosphorylation [31]. Moreover, Parashar et al. demonstrated that miR-551b upregulates IL-31RA expression and promotes breast cancer progression via STAT3-mediated transcription [32]. However, several studies have presented opposing evidence regarding IL31/IL31RA signaling in tumorigenesis [20,33]. Davidi et al. found that siRNA-mediated knockdown of IL31 increased invasion and migratory properties in colon cancer cell lines. In mouse models, IL31-depleted cancer cells grew faster than control tumors. Conversely, administration of recombinant IL31 significantly reduced tumor growth compared to controls. Furthermore, combining anti-tumor agents with human IL31-IgG compounds significantly suppressed cancer growth and angiogenesis compared to anti-tumor treatment alone [20]. Kan et al.’s study demonstrated similar results that decreased proliferation in IL31-expressing cancer cells compared to control tumors [33]. Given this evidence, the role of IL31 signaling in cancer remains controversial, and future validation studies are warranted to clarify its context-dependent effects. Moreover, although the current findings do not support IL31RA as an immediately actionable therapeutic target, our results suggest a potential association between IL31RA polymorphisms and expression patterns and the clinicopathological characteristics of OCSCC; however, this association was primarily observed in the gingival cancer subpopulation, particularly with regard to lymph node involvement. In the context of precision oncology, genetic variations in IL31RA may provide insights into interindividual differences in tumor behavior and may help generate hypotheses regarding factors associated with disease aggressiveness. Furthermore, integrating molecular findings with conventional clinicopathological parameters may contribute to a better understanding of disease heterogeneity. Nevertheless, substantial mechanistic investigations and independent clinical validation studies are required to confirm these associations and clarify the biological role of IL31RA in OCSCC progression.

Several limitations warrant acknowledgment. First, although this study included a relatively large cohort, the findings were derived from a single retrospective case–control population without an independent external validation cohort. The lack of validation in an additional, independent dataset limits the generalizability and robustness of the prognostic value of IL31RA polymorphisms, particularly regarding their role as predictors of lymph node involvement in gingival cancer. Second, IL31RA expression levels were not directly measured at either the mRNA or protein level in our patient cohort, which limited our ability to directly correlate IL31RA expression with specific allele variants. Therefore, the association between IL31RA polymorphisms, increased IL31RA expression, and nodal metastasis in our study was inferred primarily from public database analyses rather than validated using matched clinical specimens from our cohort. This limitation should be considered when interpreting the proposed genotype–expression relationship. Third, this study used of the GTEx database to evaluate genotype-related differences in IL31RA expression. However, GTEx is based on normal tissue expression profiles across multiple healthy human tissues rather than tumor-specific samples from OCSCC patients. Therefore, although the observed expression quantitative trait locus (eQTL) relationships provide supportive evidence that IL31RA polymorphisms may influence gene expression, these findings may not fully reflect regulatory mechanisms within the tumor microenvironment. Fourth, although our clinical cohort consisted exclusively of OCSCC patients, the prognostic analyses were derived from the TCGA-HNSCC dataset, which includes multiple head and neck cancer subtypes with distinct biological characteristics and prognostic factors, such as HPV status. In addition, TCGA-based expression analyses rely on bulk transcriptomic data and may be influenced by tumor heterogeneity, sample composition, and technical variability. Therefore, the observed associations should be interpreted with caution and require further validation in independent OCSCC-specific cohorts. Finally, the present study demonstrated an association between IL31RA expression and the migratory behavior of oral cancer cells. However, the underlying molecular mechanisms were not investigated and therefore remain to be elucidated. Further studies are required to identify the downstream signaling pathways and molecular networks involved in IL31RA-mediated tumor progression and lymph node involvement in OCSCC.

In conclusion, this retrospective case-control study demonstrated that IL31RA polymorphisms were not significantly associated with OCSCC development; however, the rs6876491 variant was associated with lymph node involvement, particularly in gingival cancer. Functional validation through *in vitro* experiments showed that IL31RA overexpression enhanced cancer cell migration, whereas knockdown reduced migratory capacity. Bioinformatic analyses using the TCGA, UALCAN, and GTEx databases revealed elevated IL31RA expression in OCSCC tissues compared with normal controls, and variant (minor allele) carriers exhibited higher expression levels than wild-type carriers. Furthermore, higher IL31RA expression was associated with poorer clinical outcomes. These findings support a potentially interesting association between IL31RA and lymph node involvement in OCSCC; however, further studies are required to validate these observations and elucidate the underlying molecular mechanisms.

## Data Availability

The data that support the findings of this study are available from the Corresponding Author, Shun-Fa Yang, upon reasonable request.
